# Ecologies of Repair: A Post-human Approach to Other-Than-Human Natures

**DOI:** 10.3389/fpsyg.2021.633737

**Published:** 2021-04-08

**Authors:** Gustavo Blanco-Wells

**Affiliations:** ^1^Instituto de Historia y Ciencias Sociales, Universidad Austral de Chile, Valdivia, Chile; ^2^Centro de Investigación en Dinámicas de Ecosistemas Marinos de Altas Latitudes, Valdivia, Chile; ^3^Centro de Ciencias del Clima y la Resiliencia, Santiago, Chile; ^4^Núcleo Milenio en Energía y Sociedad, Santiago, Chile

**Keywords:** environmental crises, posthumanism, relational ontology, non-human, transdiscipline

## Abstract

This conceptual paper explores the theoretical possibilities of posthumanism and presents ecologies of repair as a heuristic device to explore the association modes of different entities, which, when confronted with the effects of human-induced destructive events, seek to repair the damage and transform the conditions of coexistence of various life forms. The central idea is that severe socio-environmental crisis caused by an intensification of industrial activity are conducive to observing new sociomaterial configurations and affective dispositions that, through the reorganization of practices of resistance, remediation, and mutual care, are oriented to generating reparative and/or transformative processes from damaged ecologies and communities. Crises constitute true ontological experimentation processes where the presence of other-than-human natures, and of artifacts or devices that participate in reparative actions, become visible. A post-human approach to nature allows us to use languages and methodologies that do not restrict the emergence of assemblages under the assumption of their *a priori* ontological separation, but rather examine their reparative potential based on the efficacy of situated relationships. Methodologically, transdisciplinarity is relevant, with ethnography and other engaged methods applied over units of observation and experience called socio-geo-ecologies. The relevant attributes of these socio-geo-ecologies, beyond the individual, community, or institutional aspects, are the specific geological characteristics that make possible an entanglement of interdependent relationships between human and non-human agents. The conceptual analysis is illustrated with empirical examples stemming from socio-geo-ecologies researched in Southern Chile.

## Introduction

The Brazilian anthropologist Eduardo Viveiros de Castro is known for his Amerindian perspectivism, which is his intellectual endeavor to overcome the Eurocentric understanding of a single nature partially represented by multiple cultures. Instead, he contends that Amerindian groups propose a representational or phenomenological unity experiencing multiple natures (Viveiros de Castro, 2005, 2012). In other words, what he terms multinaturalism is the Amerindian conception of “a spiritual unity and a corporeal diversity” (Viveiros de Castro, 2012, p. 46), with the effect of an understanding of culture as the form of the universal, whilst nature would be the form of the particular. How can we go beyond an understanding of nature split by cultural representations? Or inversely, how do we experience a certain degree of cultural unity open to multiple natures if we are not (only) Amazonian Amerindians?

This conceptual paper approaches the various natures that emerge from environmental crises through a post-humanist perspective; that is, it takes full consideration of the non-human agencies shaping the world. It provides theoretical reflections on recent processes of intense damage or affectation that unleash creative social forces to rebuild broken relationships, damaged ecosystems, and obsolete institutions, through explicit recognition of the capacity of agency and practices that involve people, animals, objects, and other materials. It is precisely this network of relationships that is called *ecologies of repair*. Here, reparation is understood broadly as open-ended actions, practices, and modes of amendment of what is seen or felt as broken. It is within this process of care where life emerges with creative intensity despite destruction and ecological damage.

The heuristic notion of *ecologies of repair*^1^ is proposed to conceptually explore the ways in which different groups, in contexts of socio-environmental conflict or crisis, relate to nature, seeking to repair the damage provoked by the effects of industrial processes and transforming the conditions of coexistence for various life forms. Drawing from recent post-humanist theory (Tsing, 2015; Haraway, 2016; Braidotti and Bignall, 2018), the general assertion of this conceptual analysis is that the scenarios of severe socio-environmental crises caused by extractive industries and other destructive processes may be conducive to the emergence of new socio-material arrangements and affective dispositions that, through practices of resistance, remediation, and mutual care engender reparatory processes and/or initiatives of transformation of damaged ecologies and communities. Thus, environmental crises may constitute true processes of social experimentation in which the presence of other-than-human natures multiply and, at the same time, make the reparative agreements and attempts at governance of these disruptive phenomena more complex.

The term “other-than-human” refers to a conceptual shift in anthropology and other social sciences seeking to avoid human exceptionalism and, instead, extending the social to other entities. As expressed by Lien and Pálsson (2019, p. 4) “it signals a shift from a concern with culture and sociality as a strictly human attribute. If ‘holism’ is understood as a ‘comprehensive approach to the human condition’ (quoting Otto and Bubandt, 2010, p. 3), then a pursuit of holism in anthropology encourages us to consider the associations between humans and other-than-humans (whether they are pigs or ancestors, spirits or machines, parasites or rocks).” In this conceptual work, other-than-human natures will be used when referring to all those other entities that composed the social in an explicit understanding that human are also nature and relationally constituted. The challenges posed by reparative situations that include other-than-human natures are partially due to our limited scientific methods to explore and understand both radical ontological differences^2^ and the immanent expressions of these differences, given the contingent and indeterminate character of many of the relations that produce them.

The article is a conceptual work based on ethnographic experiences obtained through the development of extended case studies in southern Chile over 10 years. Some illustrations of my research will be provided throughout the text, as some clarifications of the theoretical intricacies may be needed. In empirical terms, posthumanism demands going beyond discrete units of analysis, moving instead toward capturing the interdependencies of a relational ontology. To achieve this goal, the analytical units of repairing ecologies are formations built over long periods that I call *socio-geo-ecologies.* By considering the geological, one can go beyond fixed political-administrative spatial units and bounded biological communities to include relevant geological attributes that are crucial to sustain the complex entanglement of relationships between human and non-human agents (more on this later).

This conceptual shift toward a more-than-human world goes beyond a cross-cultural understanding of nature and instead challenges our ability as scientists to comprehend modes of existence that destabilize the boundaries of the self and the social, the organic and inorganic, the single and the multiple, and many more deeply rooted conceptual binaries. The analysis is not centered on explaining how crises are produced, but on understanding what they produced, mainly in their dimension of ecological and sociopolitical reparation. The framework also points to the value of research designs that pay attention to ontological openings^3^. These openings are a theoretical and affective predisposition to include the heterogeneous agencies—assemblages–that flourish as situated forms and practices of reparation and re-composition of life. The concept of assemblage is key to the approach of ecologies of repair and is understood as *agencement*, following the original word of Deleuze and Guattari (1987), prior to its translation to English. An *agencement* “is an arrangement or layout of heterogenous elements” (Nail, 2017, p. 22), while the English word assemblage conveys the meaning of “a gathering of things together into unities” (Nail, 2017, p. 22). The indexical distinction is important because it expressed “the rejection of unity in favor of multiplicity, and the rejection of essence in favor of events,” (Nail, 2017, p. 22) both crucial philosophical traits to an understanding of nature without *a priori* ontological separations.

In the following sections, I present the conceptual analysis around the three lines of argument that sustain the theoretic foundation of this article: (1) crises are an opportunity for change as they open possibilities for processes of ecological reparation; (2) a post-humanist approach is necessary for exploring the interplay between ontologically diverse entities in these reparatory processes; and (3) there are theoretical and methodological challenges to practicing a post-human approach to repairing ecologies.

### Crises, Disasters, Conflicts, Ruins: Legacies of Capitalism

In the Global Assessment Report of the Intergovernmental Science and Policy Platform on Biodiversity and Ecosystem Services (Intergovernmental Science-Policy Platform on Biodiversity and Ecosystem Services, 2019), key messages from the scientific community alerting us to the dramatic state of “nature” due to the effect of humans were revealed. Approximately a quarter of the species of plants and animals are under threat of extinction, at a rate unprecedented in relation to paleo-historical records; three-quarters of the terrestrial environment and about 66% of the marine environment have been significantly altered by human actions; more than a third of the world’s land area and almost 75% of freshwater resources are now used for agricultural or livestock production; the value of agricultural production has increased by about 300% since 1970; the raw timber harvest has increased by 45%; and each year approximately 60 billion tons of renewable and non-renewable resources are extracted globally, almost double of what it was in 1980 (Intergovernmental Science-Policy Platform on Biodiversity and Ecosystem Services, 2019). The evaluation of this panel of experts, built on the review of more than 15,000 scientific publications, is emphatic in pointing out that, if significant transformative actions are not carried out in the production and consumption of energy, water, food, animal feed, and fibers, it becomes very difficult to project sustainability scenarios beyond the timeframe of 2030 (Intergovernmental Science-Policy Platform on Biodiversity and Ecosystem Services, 2019).

The alarming messages of this report, in addition to the growing concern about the materialization of the most negative scenarios of global warming has caused a shared sense of urgency that prevails in many fields of science and citizenry. The most recent report of the Intergovernmental Panel on Climate Change recognizes that it is very likely to reach a global average warming above 1.5°C between 2,030 and 2,052, with devastating consequences for biodiversity, livelihoods, food security, and water supply (IPCC, 2018). Indicative of this perception of crisis are the global mobilizations for climate actions such as Fridays for Future, the non-violent direct action campaigns of the English collective Extinction Rebellion that have multiplied throughout the planet and, the Peoples’ summits that confront, year after year, global economic forums and climate diplomacies in the Conferences of the Parties (COP). These collective actions, institutionalized through the techno-scientific world or manifested by social movements or indigenous peoples, also find vigorous expression in contemporary reflections of social scientific thought, the arts, and environmental humanities. Undoubtedly, one of the most interesting debates raised by authors working in this field of ideas is the effects that the planetary crisis, in its different meanings, is having on the conception of politics, the limits of capitalist growth, and the possible reconfigurations of culture-nature relationship.

The notion of crisis, referring to a serious deterioration of the resource base that sustains our development, has been present for more than half a century (Estenssoro, 2007). In broad terms, the literature distinguishes between ecological, environmental, civilizational, global, and ultimately climate crisis. More than simple semantic variations, their differences are important because they express the central set of relationships that would be constitutive of the crisis. They can also be indicative of the underlying ideologies and their related conceptual frameworks (Blanco and Günther, 2019). The notion of ecological crisis emerged in the 1950s during the heyday of the discipline of ecology and was conceptually massified during the environmental movements of the 1960s, through the scientific and social critique of the production systems that led to the rapid deterioration of ecosystems (White, 1967). Although many authors do not establish a conceptual distinction between ecological crisis and environmental crisis, a plausible interpretation is that the latter gained analytical strength during the succession of United Nations Earth Summits that began in Stockholm in 1972 (Estenssoro, 2007). In a certain way, the environmental crisis under the approach of multilateralism is “domesticated” in the notion of sustainable development through the gradual greening of production systems, promoted by international institutions and led by market interests (Blanco and Günther, 2019).

In stark contrast, Bartra’s idea of civilizational crisis represents a comprehensive critique of the “spatially globalizing, socially industrial, economically capitalist, culturally hybrid and intellectually rationalist order that Western civilization represents” (Bartra, 2009, p. 192). Although this critique of capitalism can be considered implicit in the previous notions, the idea of a civilizational crisis of the Western order makes it explicit that what must be radically changed is the unequal mode of material accumulation and the idea of unlimited economic growth of contemporary capitalism (Lander, 2013). By the end of the 2000s, the idea of global crisis gathered momentum with the rapid propagation of the financial crisis in the United States. Beyond its destructive economic consequences, the idea of a financial-related global crisis becomes a systemic analysis of the environmental consequences of capitalism, stemming from an intensification of extractivism and the reprimarization of the economy, with particular effects in Latin America (Gudynas and Acosta, 2011; Svampa, 2012).

The anthropogenic climate crisis and the notion of climate emergency opened new interpretations of epoch change reviving the idea of existential threats to humanity. The Anthropocene (Crutzen and Stoermer, 2000), Capitalocene (Moore, 2017), Plantationocene and Chtuluceno (Haraway, 2015), Necrocene (McBrien, 2016), Catastrophic Times (Stengers, 2015), Apocalypse (Swyngedouw, 2010; Skrimshire, 2014), Damaged Planet (Tsing et al., 2017), and End of the World (Danowski and Viveiros de Castro, 2017) become concepts-manifestos which, although politically differentiated, converge in representing a preoccupation on the reach, speed, and types of crises that modern societies will face.

The different notions about crisis and the alarming tone of global reports eloquently emphasize the magnitude of the deterioration of the conditions that sustain human and non-human life. They expound on the urgency of rethinking the relations of production, consumption, and coexistence contained in destructive capitalism, but also in the need of going beyond a non-relational way of thinking and approaching “others.” These outlooks are divided between grim, even apocalyptic prognoses and those centered on building more promising futures. The notion of crisis, in this sense, is ambivalent. On the one hand, it generates dystopias that threaten us with extinction and alienation (Diamond, 2005; Welzer, 2011); on the other hand, it creates new utopian or quasi-utopian narratives varying from technocratic approaches, stemming from planned socio-technical transitions (Geels, 2010; Giddens, 2011; Urry, 2011) to pragmatic-utopias, associated with the proposal of transformations beyond the current climatic or multilateral regime (O’Brien, 2012; Connolly, 2017; Latour, 2017), up to some optimism about the possibilities that these crises would allow us a civilizational rebirth beyond modernity (Estermann, 2012; Chandler, 2018). In this last sense, the words of Ulrich Beck resonate, when he claims we will face an “emancipatory catastrophism,” understood as the production of common goods generated from the discourses of “bad,” which would enable a “metamorphosis” of society (Beck, 2015, 2016).

Crisis, as its Greek etymology indicates, refers to a separation, to a juncture that destroys certain possibilities and, in turn, opens others. Whether as contemporaneous disastrous events or apocalyptic conceptual devices, crises are also opportunities for experimentation, not only epistemological but also ontological. An intensification of relations that provoke damage, destruction, disaster, and threat is in certain cases answered with the same intensity in the forms of resistance, sociotechnical reorganization, and proliferation of assemblages causing different forms of recomposition of life. In this sense, Anna Ting’s award-winning anthropological work is illuminating. In her book, Tsing (2015) studies the web of lives arising from livelihoods and ecologies that are precarious and damaged by capitalist means of production, in this case, articulated around the collection and international commerce of the matsutake mushroom. For Tsing, the timeline that is now called Anthropocene is associated with the advent of modern capitalism, deployed through alienation techniques, toward a humanism, which, under the illustrated idea of progress, has transformed humans and other beings into resources. These processes and techniques of anthropocentric capitalism obscure the means for collaboration for life and survival; “This ‘anthropo’ blocks attention to patchy landscapes, multiple temporalities, and shifting assemblages of humans and non-humans: the very stuff of collaborative survival” (2015, p. 20). To study this web of life, Tsing proposes a strategy she calls the “arts of noticing,” exercised through ethnography and a renewed understanding of natural history. In her words: “This kind of noticing is just what is needed to appreciate the multiple temporal rhythms and trajectories of the assemblage” (Tsing, 2015, p. 24).

The current discussions on the planetary crisis add new elements referring to the scale, the destructive effects, and the relevant actors and entities involved in the socio-environmental crises. It is not just a matter of thinking of the crisis as a general framework from which to observe specific events, but, on the contrary, of situating it both as a concrete social experience and as a heuristic device. Socio-environmental crises are, on the one hand, a state of exception that implies the deterioration of the living conditions of humans and non-human species. On the other hand, from the analytical point of view, crises constitute a heuristic device for social research. They constitute a time-space by which it is possible to observe processes of damage and recomposition that require the development of creative approaches and social experimentation. At this point, the notion of “ontological opening” is of importance. An opening is nothing other than an opportunity of maximizing the attention to the complex realities unfolding from processes of socio-environmental crises, radicalizing the enquiring strategies in an attempt to reach, sense, and care for other-than-human natures. Crisis obliges. That is why emphasis is placed on recognizing non-human agencies that, in relation to humans, are forcefully deployed in the critical processes and allow us to consider a broader composition of “the social” (Latour, 2008).

In this perspective, crisis goes beyond being a global diagnosis to becoming a field of social experience and situated experimentation that articulates different moments: from the environmental disaster, detrimental to conditions and living in a given territory, to conflict as a manifest expression of differential modes of appropriation, use, and valuation of nature among social actors, to the processes of reparation and recomposition that do not cease to be parts of the crisis.

If crises are seen as opportunities to give proper attention to other-than-human natures, it then follows the relevance of posthumanism as a necessary change in the philosophy of science and the practices of ecological reparation.

### The Post-human Turn: Theoretical Challenges for a More-Than-Human World

What has been called the post-human turn is a multiform theoretical grouping that finds affinity in the philosophical ideas of Deleuze and Guattari, particularly expressed in *A Thousand Plateaus: Capitalism and Schizophrenia* (1987). This text is widely acknowledged to be of great relevance to the development of a posthumanist ontology (Ferrando, 2019) as it gives life to a series of metaphors that evolve into concepts of social theory. The central postulates of posthumanism can be synthesized in a desire to radicalize the understanding of the assemblage of bodies and materials, the organic and the inorganic, and their mutual constitution in multiple and open forms of coexistence. Posthumanism can be conceived as an “umbrella term” that includes different theoretical lines: new materialisms, actor-network theory, theories of affect, assemblage theory, non-representational theory, speculative realism, vital materialism; and it has authors as diverse as Isabelle Stengers, Bruno Latour, Donna Haraway, Anna Tsing, Jane Bennett, Manuel De Landa, and Brian Massumi, who are among the most prominent (Wolfe, 2010; Ferrando, 2013; Grusin, 2015; Chandler, 2018). Ferrando establishes a more general differentiation between posthumanism (philosophical, cultural, and critical) and transhumanism, where both have the “common perception of the human as a non-fixed and mutable condition” (Ferrando, 2013, p. 27). Transhumanism focuses on the possibilities presented by science and technology to overcome the limits of the human in the sense of “human enhancement” which would contribute to moving toward another era. On the contrary, posthumanism is built from a radical critique of dualisms –nature/culture, mind/body, micro/macro, traditional/modern– laying the foundations to think about scenarios of coexistence in a post-anthropocentric world (Ferrando, 2013).

Posthumanism finds epistemic affinity with some of the approaches of the so-called ontological turn, which would have as a shared element, more or less explicitly, a common diagnosis: we would be witnessing a broader type of change, “the spectrum of a global ecological crisis” (Kohn, 2015) that urges us to explore possibilities for conviviality beyond the modern tools of science, democracy, and capitalism. Within the ontological turn, there is the so-called political ontology developed from the work of de la Cadena, Blaser, and Escobar (Holbraad and Pedersen, 2017), a group of Latin American authors of particular interest for this conceptual analysis. The main characteristic of political ontology is the recognition of the set of entities that populate the world in which the human, non-human, and more-than-human are heterogeneously associated, constituting “a world of many worlds” (De la Cadena and Blaser, 2018), or, in the words of Escobar, integrating pluriverses (Escobar, 2017). From this perspective, the people of Latin American territories, particularly indigenous peoples, live, name, and represent worlds, or parts of the world, ontologically different from the Eurocentric and technocratic rationality (De la Cadena, 2015). Most of the environmental conflicts occur due to the inability of institutions and modern rational epistemology to understand the incommensurability contained in certain relationships between humans and other natures (Blaser, 2013; Escobar, 2015a). Without a doubt, political ontology finds certain affinity in the critique that political ecology has established, but it also has important differences. Political ecology makes visible the underlying causes of socio-environmental crises and conflicts by examining the development of extractive industrial capitalism, the forms of appropriation of nature, and its destructive effects on territories. Although of great importance, political ecology has certain limitations because it tends to perpetuate the society-nature distinction by not decisively incorporating the agencies of non-human, more-than-human, and even inhuman entities that emerge and become visible in the conjunctures of socio-environmental crises. Thus, the material world continues to be subordinated to social relations, the latter being understood as a capacity exercised only by humans.

Posthumanism also supposes, as Braidotti points out, the problematization of those positions that assumed that humanism expresses a condition of equal belonging to the same category: we are not all equally human (Braidotti, 2017). Humanism is not and has not been a category of universal neutrality. In its normative dimension, it has been revealed to us as an accomplice of violent exclusions toward those considered less-than-human: women, non-heterosexuals, people of color, the disabled, and indigenous people (Roffe and Stark, 2015).

The idea of ecologies of repair finds affinity with the political ontology scholarship and partial coincidence in a book recently published and edited by Braidotti and Bignall (2018) entitled *Posthuman Ecologies*, in which several authors examine the scope of Deleuze’s philosophy on a posthumanist and feminist agenda. For Bignall and Braidotti (2018, p. 1):

“[t]he ‘posthuman turn’ defined as the convergence of posthumanism with postanthropocentrism- is a complex and multidirectional discursive and material event. It encourages to build in the generative potential of the critiques of humanism developed by radical epistemologies that aim at a more inclusive practice of becoming-human. And it also supports and opening out of our conceptual imagination, the power (potentia) of thinking beyond the established anthropocentric frame toward becoming-world.”

Ecologies of repair finds important theoretical resonances with the work of these authors, whose purpose is oriented to a broad and ambitious philosophical reflection, but it differs in two main aspects. First, it pays particular attention to the empirical experience. The possibility of understanding repair processes in crisis contexts from a sociomaterial perspective supposes a methodological predisposition to go beyond the representational (Vannini, 2015), maintaining the principle of situationality that allows qualitative social research for the study of emergent processes between bodies, materials, affects, relationships, and events. Second, taking into account a criticism made of the teleological orientation of certain posthumanist approaches (Grusin, 2015), this proposal does not conceive posthumanism as a starting point for overcoming the human. A posthuman-centered approach does not in any way imply a new separation of humans, non-humans, and more-than-humans into independent domains, in which the latter two appear on the scene to overthrow the former. On the contrary, it is precisely about exploring their interdependencies in inter and multi-species relationships (Haraway, 2003; Kohn, 2013; Kirksey, 2014), in their symbiogenetic manifestations (Margulis, 1998/2008), and sympoietic creativity (Haraway, 2016) in mutual constitution with the inorganic world. The latter does not only mean taking into account what these materials afford us (Ingold, 2000, 2011), but also consider their infrastructural dimensions (Jensen and Morita, 2015), their seismic consequences (Farías, 2014), the unreachableness of geological entities (Tironi, 2019), and, more generally, the geontological distinction that emerges between Life and Non-Life (Povinelli, 2016), which cannot be reduced exclusively to human experience or that of other living beings. Beyond their differences, these investigations highlight the ontological excess always present in the tensions of nature, that is, the impossibility of reducing to “things” –passive materials that only decorate the material background of social life– the multiple entities that enliven, suffer, and confront socio-environmental crises.

In order to illustrate what I am referring to with these post-human ecologies facing critical events, I provide a brief ethnographic vignette stemming from my research program in one of the socio-geo-ecologies under study: the Rio Cruces Estuary in southern Chile.

### Dying Swans: An Ethnographic Vignette

In the spring of 2004, a video of a black-necked swan, floating in the water with difficulty and that could barely hold its head up began to circulate in Chilean news. This image and others of dead and extremely debilitated swans were captured by citizens of Valdivia in the Rio Cruces Estuary in southern Chile, who, worried about the situation, begin to patrol in boats and small airplanes^4^. In one of these videos the renowned ornithologist and researcher who had studied the wetland for decades, Professor Roberto Schlatter, noted the absence of birdlife with dismay, “we are in a silent spring, like Rachel Carson wrote about when birds died because of pesticide application…they no longer sang.”^5^ Testimonies multiplied. Swans began to fall in patios, on rooftops, were starving or dead. The same thing began to be seen with coots, ducks, and coypus. Scientists began to speak of the abrupt and massive death of *egeria densa*, a waterweed known as luchecillo, of which the birds feed off, as a possible explanation. The people of Valdivia and neighboring towns that lie by the great wetland went from being concerned to indignation. Manifestations began. People pointed to the only condition that had changed during the beginning months of 2004: In January of that year, upriver, in the northern area of the Cruces River, the CELCO cellulose plant became operational. Nine years later, in 2013, a civil court judge, in an unprecedented ruling in Chile, found the company guilty of environmental damage and required it to finance a five-point, long and medium-term, reparatory process. The company abided by the ruling, and the Social Scientific Council was created to define how to implement these measures. This was a novel body with public, private, and civil society participation, with the aim of establishing general guidelines for compliance. This point of judicial inflection marks the beginning of various reparation and conservation processes that have taken place to this day. Although industrial pollution and other emerging threats have not faded, the swans seem to thrive once more in the Cruces River. What processes and entities have operated and are operating to achieve this change in the state of affairs?

The environmental damage in the Cruces River wetland is not unique in Chile or other parts of the world. Ecological disasters and socio-environmental crises have become common in recent history and particularly intense over the last two decades, in the scale of damage as in the strength of collective actions responding to them. Faced with these processes of threats to life, questions arise: What is reparation in contexts of severe socio-environmental damage? How can we understand the forms and entities that participate in reparatory processes beyond those of institutional governance? How are social sciences implicated in these processes? What is nature and how does it become manifest in the contexts of crisis?

### Ecologies of Repair: An Approach for Experiencing Multiple Natures

The notion of ecologies of repair offers a posthumanist perspective oriented to the situated understanding of processes in which the constituencies of culture and nature are reconfigured by the effect of heterogeneous associations between humans and non-humans and therefore, their boundaries are challenged and eventually dissolved. This perspective does not ignore the asymmetries between human groups and between those with other non-human entities, but rather adopts the epistemological position of not establishing *a priori* hierarchies, precisely with the intention of empirically reconstructing how these differences and exclusions are produced (Hetherington and Munro, 1997). This recognition, the practical effects of the power of hierarchies and binary divisions, is a starting point where we can open ourselves up to feel, think, and experience the multiple natures, the relationships between the infinite entities that compose it, and imagine the coexistence in a more-than-human world.

Under this general orientation, it is assumed that severe socio-environmental crisis caused by the effects of voracious capitalism are conducive to observing new sociomaterial configurations and affective dispositions that, through the reorganization of practices of resistance, remediation, and mutual care, are aimed at generating reparative and/or transformative processes from damaged ecologies and communities. Repair, in the sense attributed here, finds quite an affinity with the notion of care defined in the work of María Puig de la Bella Casa as a “mode of attention to a more than human life-sustaining web” (Puig de la Bella Casa, 2017, p. 217). To repair as to care –sensu Puig de la Bella Casa– is not conceived from a moralizing point of view, nor exclusively from a naive affectivity. Repairing, from a post-humanist perspective, supposes that we decenter our gaze from the possibilities exercised by human agency to notice what emerges as regenerative possibilities from the entanglement of life forms in a specific space-time. This also means understanding the limits of relationality –for example, in the face of a destructive force of an extreme climatic event, the annihilating contamination of a pulp-mill plant, an oil spill, or the toxicity of mining tailings (Ureta and Flores, 2018). It also implies recognizing what cannot be repaired, what has been broken forever, the finiteness of life and, in more extreme but possible cases, extinction^6^. The idea of things that cannot be repaired is an important one, as it makes itself evident when stated by a Mapuche professional commenting on the wetland case presented in the ethnographic vignette. She was a young girl when the crisis caused by the pulp-mill plant unfolded and believes that there are things that cannot be repaired, such as the destruction of places, the death of animals, but more importantly, the trust among neighbors, in reference to the divisions created by the company’s presence.

Where can we observe these ecologies in repair? The possibilities are unfortunately vast, as witnessed by the accelerating deterioration of ecosystems and the devastating consequences of the climate crisis. However, the analytical unit proposed to study reparatory processes in/with other-than-human natures are not individuals, groups, communities, or institutions, something that seems counterintuitive for some social sciences, but what I have called *socio-geo-ecological formations*. This unit of observation and *experience* is proposed to highlight that, over socio-demographic, political, or administrative aspects, its most relevant attributes are concurrent with the particular geological characteristics of a place on which a complex network of interdependent relationships between human and non-human agents, built over long periods, are sustained. For example, in the case of my research these socio-geo-ecologies are an estuary, a salt flat, an archipelago and an island. Currently, a large part of these formations presents the common experience of having been affected by different types of critical events. They have been exposed to severe damage by different forms of environmental deterioration or abrupt changes in socio-ecological relationships, as consistently demonstrated by diverse global assessment reports, such as The Global Environment Outlook (UN Environment, 2019), the Global Assessment Report on Biodiversity and Ecosystem Services (Intergovernmental Science-Policy Platform on Biodiversity and Ecosystem Services, 2019), and The Living Planet Report (WWF, 2020).

The possibilities of the empirical study of these damaged socio-geo-ecologies can multiply, as in the Latin American case, due to the development of extractive industries that have positioned themselves in the production of commodities or production resources, such as minerals and energy, key to national or global exporter processes, but in other latitudes, they may be linked to other destructive processes, such as wars, accelerated urbanization, and nuclear disasters, among others.

In these socio-geo-ecological formations, the methodological focus must be centered on the reparatory, transitional, resistance, and existence processes that different entities, present or emerging, carry out. In other words, methodology is conceived as an openness and willingness to maximize the experience and exposure to these socio-geo-ecologies, increasing our ability to notice the assemblages that unfold around and after critical or disruptive events. The processes that we call reparatory can manifest themselves in different ways: as novel organizational arrangements, as new forms of inter or multi-species interaction, as forms of reconciliation between production, self-reliance, and consumption, as preservation and restoration actions, as forms of healing and self-care, as expressions of artistic creation, etc^7^. This list is, of course, not exhaustive. It will be through the empirical evidence of case studies and the narration of concrete experiences that the value that the agents attribute to different actions, practices, relationships, and entities of what we call ecologies of repair can be determined. For example, in the aftermath of the vignette presented above, reparation is expressed in many ways: through on-site courses held at the newly created Río Cruces Wetland Research Center that have increased the number of locally trained birdwatchers; through communitarian programs aiming at the reconstruction of river docks in effort to recover lost practices of navigation (displaced for many years due to the construction of a highway); and also through newly discovered interspecies interactions as when young sea-lions predate black-necked swans up-river, creating a conservation dilemma for local people given that both species are protected. All three examples have implied the setting of more-than-human alliances, between scientist, birds, local school students, park-rangers, local authorities, sea-lions, etc.

The scale of observation of these processes, normally organized hierarchically by the anthropocentric institutional focus, can also be subverted when the unit of analysis is socio-geo-ecological formations. In this perspective, every scale matters, because from a perspective focused on interdependent relationships, these formations are made up of different communities, from those microscopic and invisible to the human eye, to larger aggregations such as forests, wetlands, monumental geological structures such as a mountain, or human-built structures such as cities. All these scales of interaction and interdependence present complex, multi-species relationships that are constitutive of the processes that sustain life. Escobar (2018) develops this idea of multiple and nested scales when he refers to his vision of pluriversal transitions, which can be understood in relation to the repair processes indicated here: “This conceptualization endows transition visions with a scalar imagination that avoids the conventional vertical hierarchy of scales, which inevitably gives too much weight to the global and too little to the local or place-based… Thinking in terms of nested structures and networks provides the basis for a distributed understanding of agency” (Escobar, 2018, p. 156).

How can we observe and experience these ecologies in repair? Considering the movement from the epistemological to the ontological, the challenge consists of moving the disciplinary limits toward experimental and not exclusively representational forms of transdiscipline. As Puig de la Bella Casa points out:

“The thinking at stake is transdisciplinary to the core, involving a wide range of perspectives and methodologies in the social sciences and humanities that form also relatively new fields: science and technology studies, animal studies, posthumanist philosophy and ethics, environmental humanities. The cultural, political, and ethical challenges are colossal and the search for alternatives ongoing” (Puig de la Bella Casa, 2017, p. 12).

The methodologies to experience multiple natures in repairing ecologies should point to radicalizing our affective disposition toward a more-than-human world –Tsing’s arts of noticing– and the ways an interdependent web of life is made possible and thrives in specific socio-geo-ecological formations. These multi-species communities, when observed beyond the exclusively human institutions and agencies, carry out reparation actions and recomposition of the damages caused by extractive, polluting, or destructive activities characteristic of capitalist modes of production.

Paradoxically, as Povinelli’s (2016) conception of geontology suggests, is the interaction with the Non-life fraction of the world that enables the development of life, but also put an end to it. In the first sense –Life and Non-Life entanglements as life enabling– let us think, for example, of the mineralization processes through which biotic communities of fungi, plants, and animals are constituted, literally, thanks to our association with the rocky fraction of our world. Thus, geology is not only a layout of inert materials outside our bodies as minerals are a constitutive part of the tissues and skeletons of what we called the living. This “geological infiltration,” as Manuel De Landa calls it (De Landa, 1997, p. 27), is one of the processes undergone by human and other species since the beginning of life on Earth. In the second sense, –Life and Non-Life entanglements as life ending– can be illustrated by the processes of air pollution, tailings, oil spills, nuclear radiation, and others that have the power to damage or kill cells, organs, bodies, and ecosystems. It is in this sense that, conceptually and empirically, socio-geo-ecological formations are not equivalent to an inorganic layout, a landscape, or a life-supporting system operating as a passive scenario of different entities’ lives. A socio-geo-ecological formation *is* the entanglement between the organic and inorganic, the material and the social, the micro and the macro, the single and the multiple, in radical opposition to analytical binaries.

Methodologically, a post-human approach to other-than-human natures should be less concerned with representing the world than with maximizing the sensual and imaginative experience of the many unfolding worlds. Emphasis on experience and engagement is related to ethnography or other methodological approaches that allow for the understanding of this ontological dimension from a practical, non-essentialist perspective, at the same time recognizing that whoever exercises the description and conceptualization of these formations participates in their reinvention (Gad et al., 2015). This is what Christopher Gad and co-authors call practical ontology, to convey the idea that those participating in research processes ethnographically are not only representing worlds but constitute them through practical and material engagements. These authors explain this idea:

“This is why anthropology must proceed *as if* there are many worlds. Studies of practical ontology can only move forward on the hypothesis that there are many worlds. Rather than making a choice between ‘multi-culture’ and ‘multi-nature,’ such studies thrive on the exploration of never-finally closed nature-cultures; the crystallization of specific ontological formations out of infinitely varied elements” (Gad et al., 2015, p. 83).

Beyond the aptitude of ethnography –and of anthropology as a discipline that used to monopolize it– is the empirical, embedded, embodied, and engaged study of crisis and repair processes, which enables a methodological opening toward agencies and entities that other human-centered methodologies ignore. This predisposition to be affected by more-than-human encounters provides a certain post-human reflexivity to the research, which contributes as an epistemological alert to avoid the anthropocentrism contained in an important part of the methodologies of the social sciences. In this direction, it is also essential to incorporate other expressions of those encounters that we call methodological, such as events, performances, walks, and the imaginative use of materialities such as photographs, artworks, and objects that allow for the presentation of extra-linguistic dimensions of human and non-human interactions in the socio-geo-ecological formations studied.

The empirical reconstruction of repaired ecologies from a post-human approach allows us to use languages and methodologies that do not restrict the proliferation of entities under the assumption of their *a priori* ontological separation, but rather examine their potential based on their expression in emerging assemblages. This will allow for a situational understanding of the experiences of crises, ruptures, deterioration, and damage by various agents, as well as forms of collective re-composition and re-articulation to overcome them, creating spaces for action and life, beyond corporate interventions of both companies and state.

## Conclusion

In this conceptual analysis, I have presented crises as an opportunity for transformation in which other-than-human natures may play a central role in processes of ecological reparation. A greater consideration of posthumanist theory is needed to advance in a relational view of nature and the attention to assemblages that emerge from damaged or disrupted socio-geo-ecologies. The approach I called ecologies of repair, may be a way, among others, to understand the different views about what nature is, beyond perspectives of multicultural representations and beyond the ontological specificities of Amerindian multinaturalism.

Crisis and reparations are dynamic processes; they do not occur as isolated and contained events but are constantly evolving. In some cases, these crises are diluted in the continuity of daily life or go unnoticed due to the lack of media attention. In others, they become central to the reorganization of human and non-human experience and existence. While these conclusions are being written, news has arrived from the Rio Cruces Wetland, which was presented in the vignette: In certain sectors, the *luchecillo* (a water weed) seems to be in trouble again. It is turning brown and seems damaged. This time the aquatic plant triggers the deployment of a set of agents more quickly. Scientists are mobilized, sampling water and aquatic plants. Observations of birds, mammals, and fish are intensifying, and the human communities surrounding the wetland are alert. The assembly between crisis and repair is reconfigured this time more quickly to respond with greater attention to this web of life we call a wetland. Perhaps repairing is precisely about increasing that capacity to notice with greater sensitivity that more-than-human world to also understand the possibilities and limits of our own humanity. Repair, in this sense, approaches its etymological origins of preparing or getting ready again. This new disposition implies recognizing and appreciating the multiple more-than-human ecologies that we call nature. Nature, in this sense, can be conceived as situated experience between multiple entities entangled in processes that enable or limit life.

The theoretical explorations that posthumanist philosophy offers have important consequences for scientific practices and major challenges for the social sciences since they force a post-anthropocentric movement, which to a certain extent, invites us to place ourselves beyond the individual, the community, society, and other social categories that we have long embraced. Planetary crisis, this time conceived as ontological openings, perhaps do give us the freedom to travel new paths or return to places that we have abandoned.

## Author Contributions

GB-W prepared and wrote the manuscript, it is based on a review and synthesis of knowledge from multiple disciplines and sources including his own research and experience.

## Conflict of Interest

The author declares that the research was conducted in the absence of any commercial or financial relationships that could be construed as a potential conflict of interest.

## Footnotes

1 In this paper, ecologies of repair and repairing ecologies will be used interchangeably.

2 In this work, radical ontological differences can be understood as both: a very different entity from my own humanity or a very similar one, as in the work of Viveiros de Castro. Drawing on his studies about shamanism, he contends, “for Amazonian peoples, the original common condition of both humans and animals is not animality but, rather, humanity” (Viveiros de Castro, 2004, p. 465).

3 de la Cadena (2014) introduces the notion of ontological opening in order to move away from the idea of “ontological turn,” as this aperture, beyond a mere academic fashion, will allow for the questioning of modern policy in its exclusive selection of those entities that may be subject to public action. Claudia Sepúlveda, in her thesis on the disaster of the Rio Cruces Nature Sanctuary, defines ontological opening “as the state of suspended and fractured reality resulting from events that drastically interrupt the ‘normal state of affairs.’ In so doing, such events slow down thought while making visible the agencies, workings, and investments involved in such a state, eventually exposing these to public revision and confrontation” (Sepúlveda, 2016, p. 8).

4 These images were produced in 2, 8 min videos by the local veterinarian Daniel Boroschek and circulated on social media under the title “Tragedy of the Swans, Valdivia Chile.”

5 Excerpted from “Tragedy of the Swans Valdivia, Chile” Part 1. Daniel Boroschek. See at: https://www.youtube.com/watch?v=GkjoeECzmc0&t=233s.

6 Tom van Dooren develops an interesting definition of extinction to avoid the nature/culture dichotomy in favor of a relational view of species –human and others– woven together in conforming multi-species communities: “From this perspective, extinction takes the form of an unraveling, a breakdown of existing patterns of relationship. Whether it be the ‘ecological’ loss of a pollinator or seed disperser or the ‘cultural’ breakdown of a funerary system –in a world that is inherently relational, absences cannot help but bring about unravelings” (van Dooren, 2018, p. 172).

7 This list of regenerative possibilities has a certain affinity with the interpretation that the Colombian anthropologist Arturo Escobar has given to the notion of design, in his recent works on transitions: “pluriversal ontological design is aimed at enabling the ecological, social, and technological conditions where multiple worlds and knowledges, involving humans and non-humans, can flourish in mutually enhancing ways” (Escobar, 2015b).
